# A Rare Case of an Intrapulmonary Teratoma Masquerading as a Pulmonary Hamartoma

**DOI:** 10.7759/cureus.113945

**Published:** 2026-08-04

**Authors:** Hamza Azal, Mouhsin Ibba, Joel Bayem Cedric, Hicham Fenane, Yassine Msougar

**Affiliations:** 1 Department of Thoracic Surgery, Mohammed VI University Hospital, Marrakesh, MAR; 2 Faculty of Medicine, Cadi Ayyad University, Marrakesh, MAR

**Keywords:** germ cell tumor, hamartochondroma, intrapulmonary teratoma, lobectomy, lung mass

## Abstract

Teratomas are fully formed germ cell tumors derived from multiple germ layers. While most commonly gonadal, they can occur in extragonadal sites, particularly the mediastinum. Isolated intrapulmonary teratomas without mediastinal extension are exceptionally rare, with fewer than 100 cases reported in the literature, and can be mistaken for other calcified lung lesions such as hamartomas.

We report the case of a 57-year-old former smoker who presented with a three-month history of chest pain and dry cough. Chest X-ray and computed tomography (CT) scan revealed a large, well-circumscribed left lower lobe mass with central "popcorn" calcification, initially suggestive of a pulmonary hamartoma. Tumor markers were negative, and all other investigations were within normal limits. The patient underwent a left lower lobectomy via posterolateral thoracotomy. Histopathological examination revealed mature cartilaginous, adipose, bony, nervous, and glial tissues without any immature or malignant component, confirming the diagnosis of a mature intrapulmonary teratoma. The postoperative course was uneventful.

Intrapulmonary teratoma, though rare, should be included in the differential diagnosis of calcified lung masses mimicking pulmonary hamartoma. Complete surgical resection remains both diagnostic and curative, with an excellent prognosis for mature forms, though long-term clinical and tumor-marker surveillance is warranted.

## Introduction

Teratomas are rare germ cell tumors composed of mature or immature tissues derived from one or more of the three embryonic germ layers (ectoderm, mesoderm, and endoderm). Although the gonads are the most common locations, they can also be found in other extragonadal sites, most notably within the thoracic cavity [1,2].

From a topographic standpoint, intrathoracic teratomas are classically situated in the anterior mediastinum. Their occurrence entirely within the pulmonary parenchyma, without any mediastinal component, is exceedingly uncommon; fewer than 100 such cases have been documented in the world literature to date. We report the case of a 57-year-old man referred for chest pain related to a large intrapulmonary mass, in whom surgical resection established the diagnosis of a mature teratoma [3].

## Case presentation

A 57-year-old male former smoker with a history of 30 pack-years who ceased smoking one year ago presented to his local hospital complaining of a three-month history of chest pain and dry cough. His past medical history was unremarkable.

**Figure 1:**
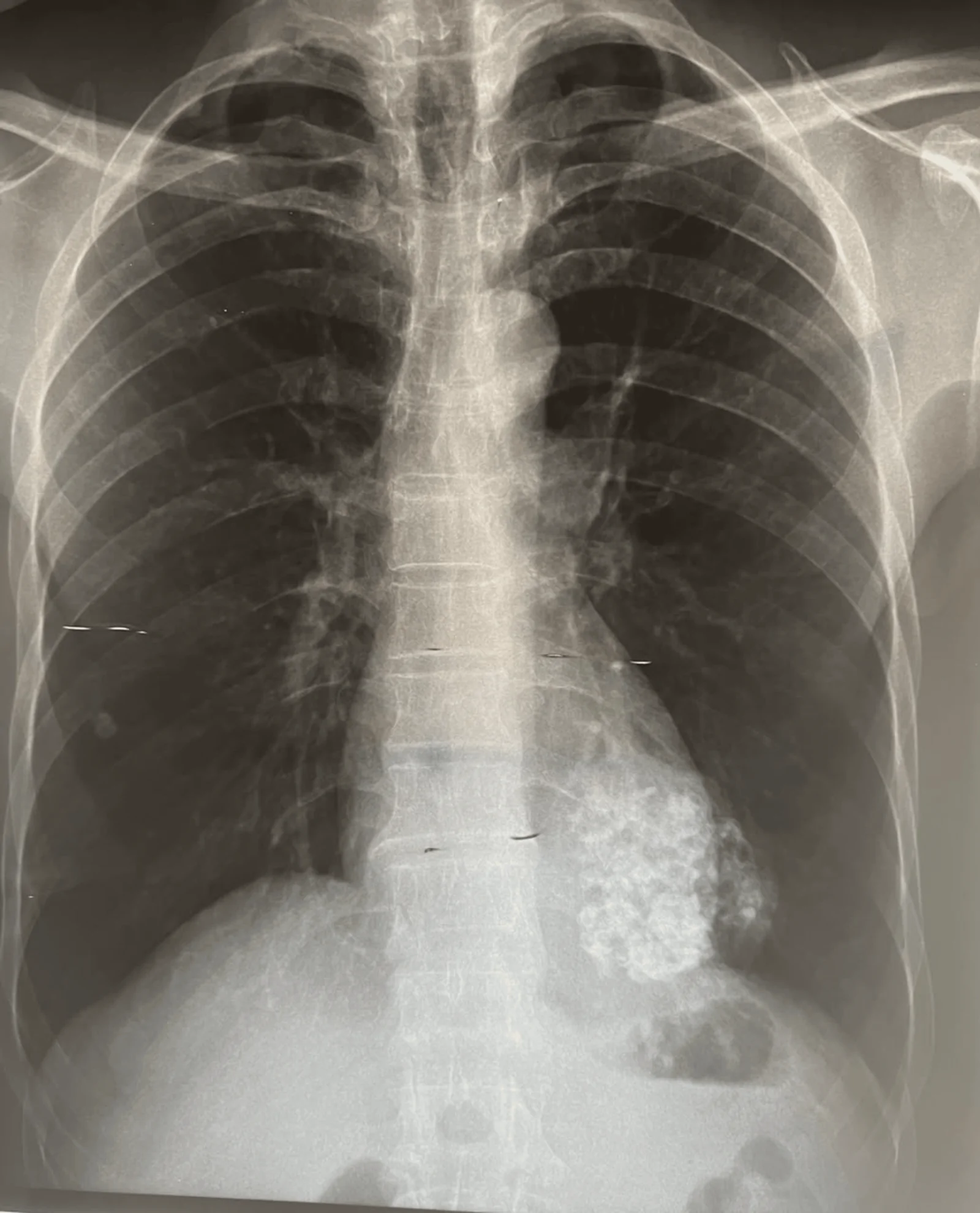
A well-defined margin, heterogeneous soft-tissue density opacity with central calcifications is noted in the lower third of the left lung field, in the posterior paracardiac area, with a negative silhouette sign. A well-circumscribed calcified nodule is noted in the lower half of the right lung field.

Chest X-ray revealed a well-circumscribed, heterogeneous mass in the left lower zone (retrocardiac region). The lesion contained prominent, dense, coarse calcifications mixed with radiolucent areas. The mass partially overlaps the cardiac silhouette and left hemidiaphragm. A tiny calcified nodule is also noted in the right lower lobe. The lung parenchyma, except for the mass, appears normal. The bony thorax reveals no abnormality (Figure 1).

**Figure 2:**
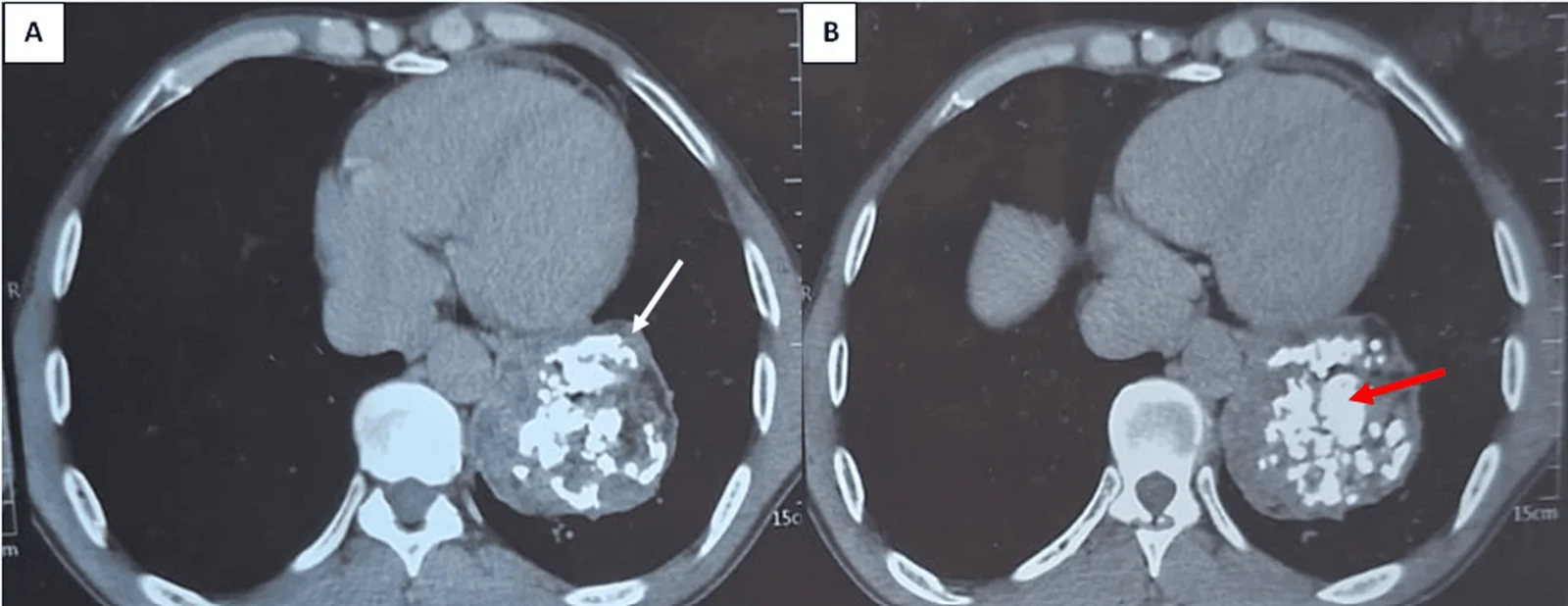
(A-B) A large proximal mass of the lower lobe of the left lung (white arrow) with central popcorn calcification (red arrow).

Computed tomography (CT) scan of the chest revealed a well-circumscribed, large, rounded mass in the retrocardiac region of the left lower lung field. The mass demonstrated extensive, prominent internal popcorn-like calcifications with areas of low soft-tissue attenuation and fat density, suggestive of a pulmonary hamartoma (Figure 2).

The patient was referred to Mohammed VI University Hospital Center of Marrakech for further workup.

Upon admission, a comprehensive biological assessment was conducted, including tumor markers (alpha-fetoprotein and beta-hCG), complete blood count, kidney and liver function tests, and serum electrolytes, all of which were within normal limits. Sputum analysis was negative for tuberculosis (Table 1).

**Table 1:** Laboratory results from the various tests performed. AFP: alpha-fetoprotein; BHCG: beta-human chorionic gonadotropin; ALAT: alanine aminotransferase; ASAT: aspartate aminotransferase; MTB: *Mycobacterium tuberculosis*

Analysis	Results	Normal Values
AFP	6 ng/mL	<6-10 ng/mL
BHCG	0.4 mIU/mL	<2 mUI/mL
Hemoglobin	13.6 g/dL	13-17 g/dL
Leukocyte	4260/µL	4000-10000/µL
Urea	0.38 g/L	0.19-0.45 g/L
Creatinine	10.28 mg/L	7-12 mg/L
ALAT	13 U/L	<55 U/L
ASAT	27 U/L	5-34 U/L
Sodium	141 mmol/L	135-145 mmol/L
Potassium	4.18 mmol/L	3.5-4.5 mmol/L
GeneXpert	MTB not detected	

**Figure 3:**
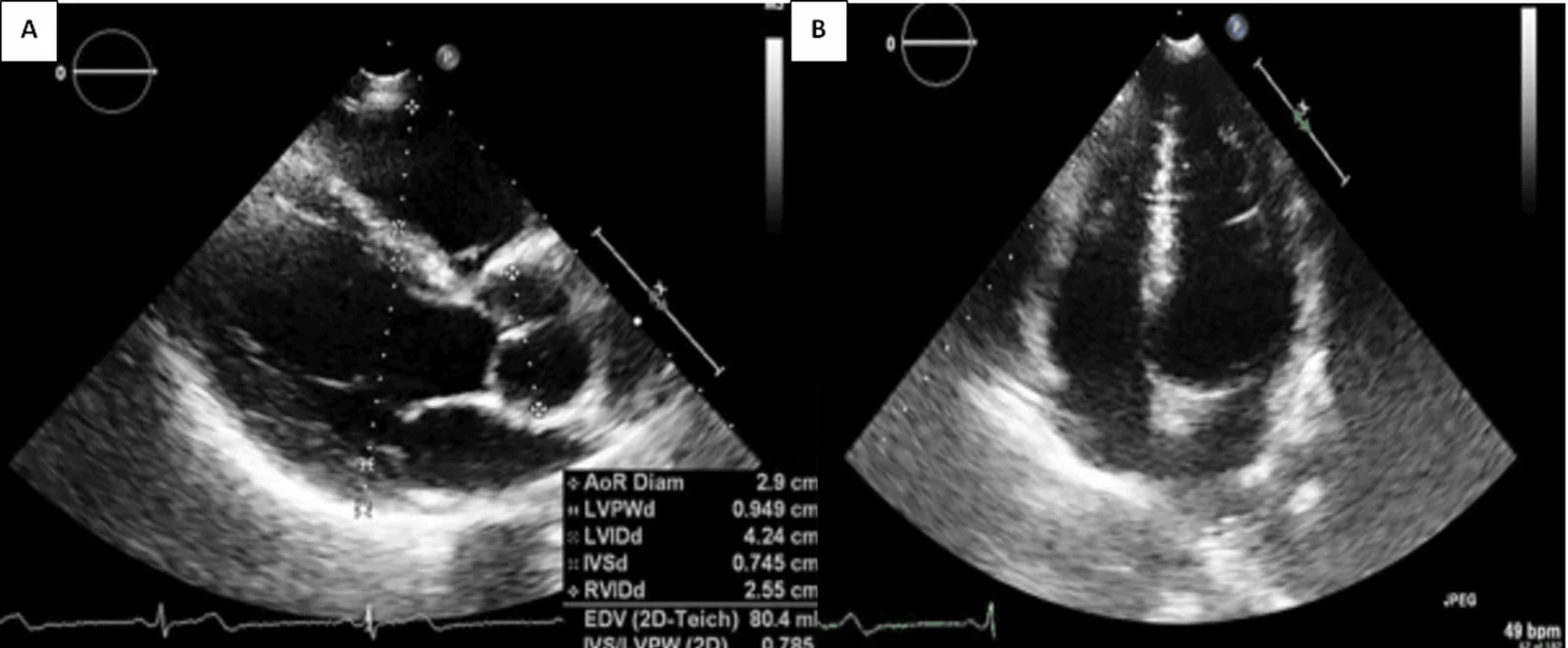
Two-dimensional transthoracic echocardiogram. (A) Parasternal Long-Axis View: Demonstrates normal left ventricular (LV) structure and systolic function. No pericardial effusion or signs of cardiac compression/tamponade are observed. (B) Apical 4-Chamber View: Confirms preserved left ventricular ejection fraction (LVEF). No intracavitary masses, thrombi, or tumors are visible in any chamber.

CT scan of the brain, abdomen, and pelvis revealed no abnormality. Lung function tests were within normal limits. Transthoracic echocardiography (TTE) showed normal cardiac function with no evidence of pericardial effusion (Figure 3).

**Figure 4:**
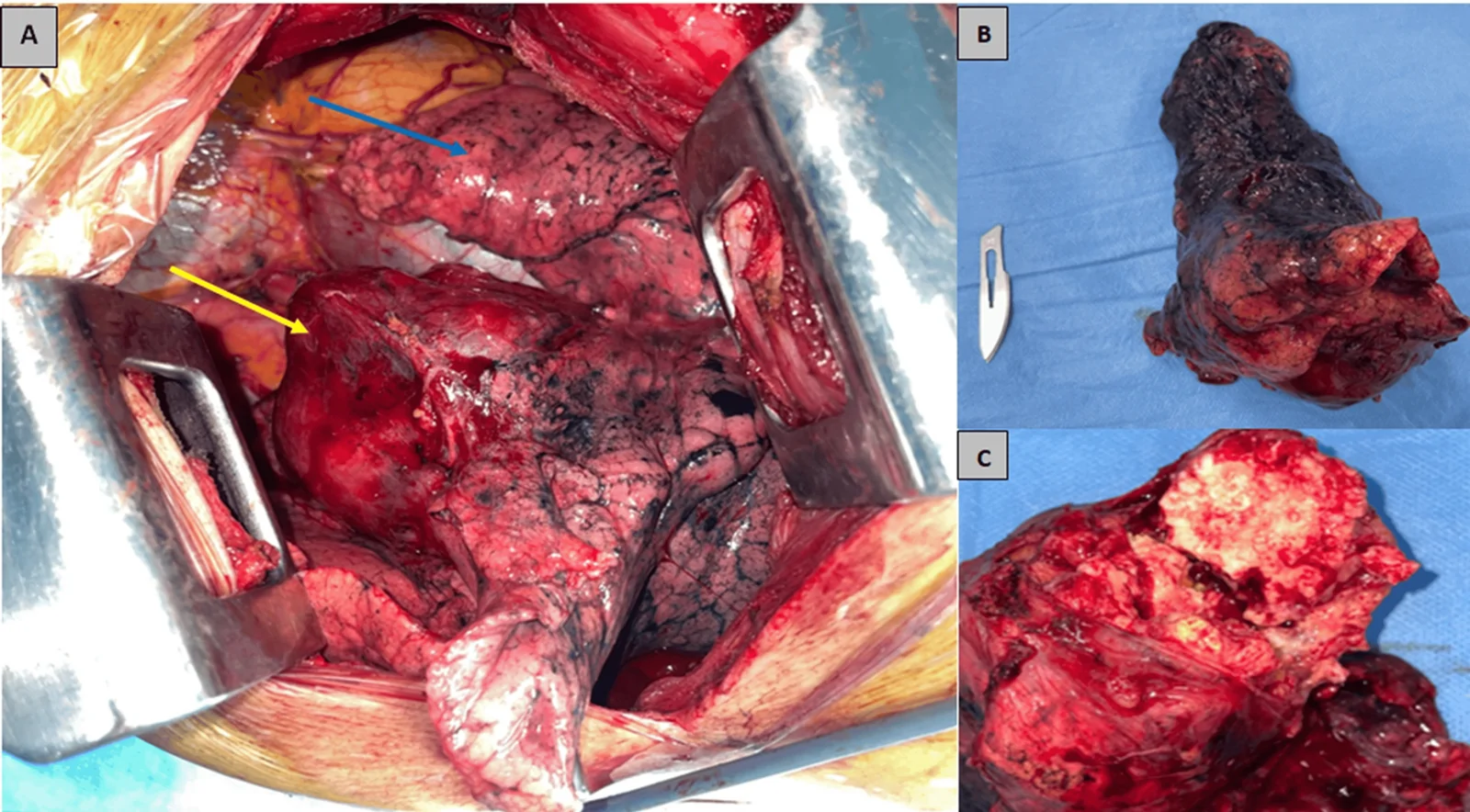
(A) Intraoperative view of the mass in the left lower lobe (yellow arrow). The healthy left upper lobe is indicated by the blue arrow. (B) Resected specimen of the left lower lobe. (C) Cut section of the mass from the lower lobe specimen showing macroscopic findings of firm, yellowish, solid tissue inside the lesion.

The left thoracic cavity was approached through a left posterolateral thoracotomy through the sixth intercostal space. A large, firm, lobulated mass was found in the left lower lobe. The mass was well demarcated, with no adhesion to the pericardium or aorta noted. A left lower lobectomy was performed. The remaining left lung expanded well after the operation (Figure 4).

**Figure 5:**
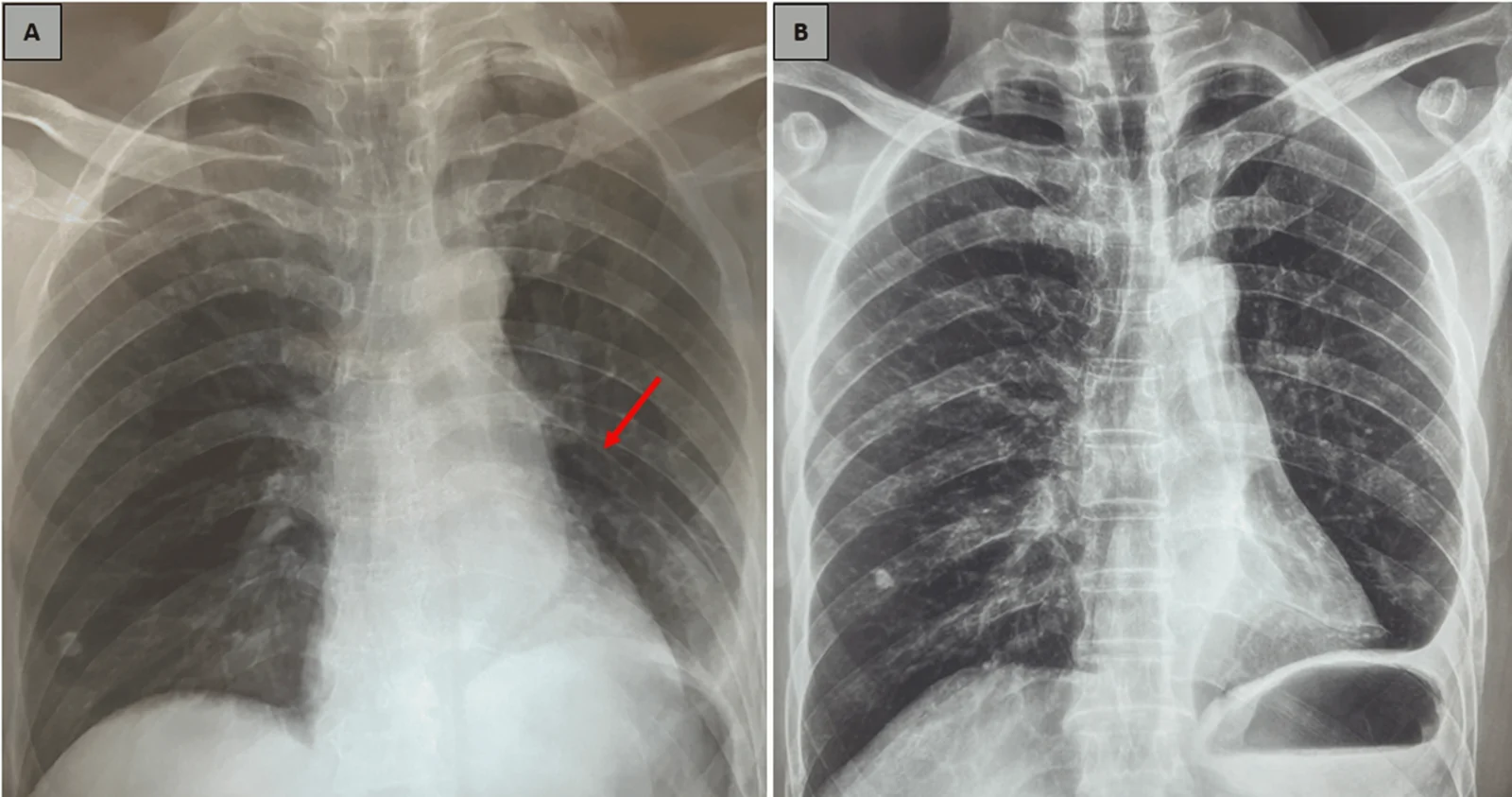
Chest X-ray. (A) Revealed re-expansion of the lung adhered to the wall, with the drain in place (red arrow). (B) Chest X-ray one month later.

The patient spent one day in the intensive care unit (ICU) in the immediate postoperative period, followed by three days on the ward before drain removal and discharge. The patient demonstrated a positive clinical, biological, and radiological (Figure 5) recovery trajectory and was subsequently given an appointment one month later for further management and monitoring of the tumor markers (alpha-fetoprotein and β-human chorionic gonadotropin), which were within normal limits.

**Figure 6:**
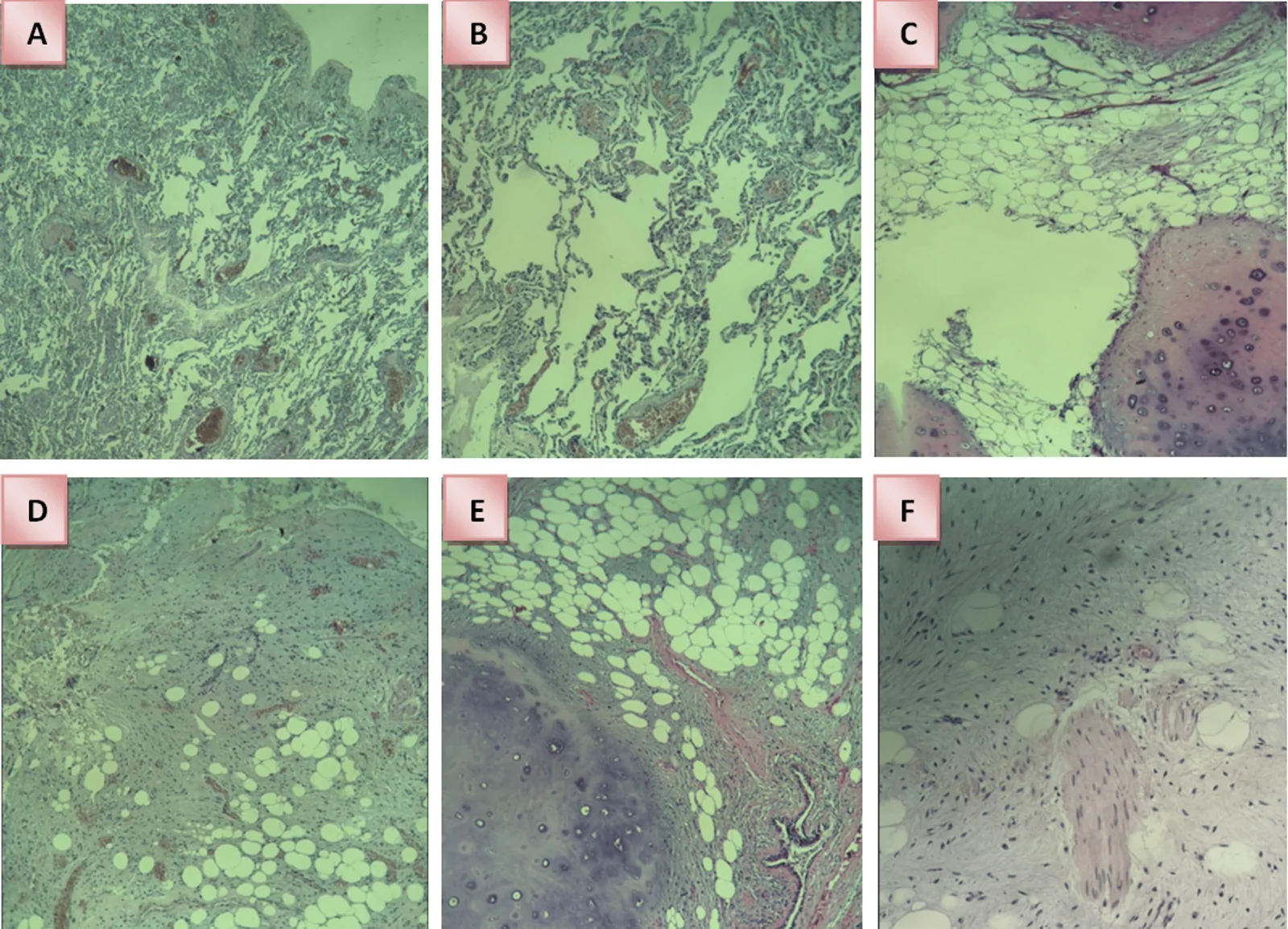
Histopathological images. (A) Low magnification (×40): adjacent lung parenchyma with preserved alveolar architecture and vascular structures. (B) High magnification (×100): adjacent lung parenchyma with preserved alveolar architecture and ectatic vascular structures. (C) Low magnification (×40): mature hyaline cartilage bordered by mature adipose tissue within the lesion. (D) Low magnification (×40): a combination of mature hyaline cartilage, mature adipose tissue, and fibro-connective stroma, illustrating the tissue diversity of the lesion. (E) Low magnification (×40): a combination of mature hyaline cartilage, adipose tissue, and a bronchial structure lined with respiratory epithelium. (F) Overview showing a fibro-connective stroma containing mature adipose tissue and bundles of smooth muscle tissue.

Histological examination reveals lung parenchyma containing ectatic bronchial structures, with focal areas of hemorrhagic content surrounded by moderate fibrosis. These bronchial structures are lined by respiratory epithelium, which is frequently ulcerated and replaced by polymorphic granulation tissue. Additionally, various mature elements, including cartilaginous, bony, adipose, nervous, and glial tissues, are present without cytonuclear atypia or immature malignant components (Figure 6). The histopathological findings are consistent with a benign mature teratoma. 

## Discussion

Teratomas originate from pluripotent stem cells and are characterized by the presence of tissue types foreign to their anatomical site of origin. Their behavior is most often benign, although malignant transformation remains possible. The intrapulmonary location, which is particularly rare, is thought to result from the ectopic migration of primitive thymic tissue along the pathway of the developing lung bud during organogenesis, a hypothesis supported by the identification of thymic remnants in some resected specimens. Reported ages range from 10 months to 65 years, with a predominance in children and adolescents and no notable difference according to sex [4].

Intrapulmonary teratoma may be asymptomatic and found incidentally on a chest radiograph, or it may present with cough, hemoptysis, chest pain, dyspnea, and trichoptysis [4]. Additionally, the tumor may present with potential complications related to the pressure effect, such as atelectasis, bronchiectasis, and postobstructive pneumonia [5]. Trichoptysis (coughing up hair) is a rare but highly characteristic finding seen in roughly 11% of cases, which reflects a fistula between the tumor and the bronchial tree [4]. In our case, chest pain was the only manifestation, with no other associated symptoms.

Radiologically, intrathoracic teratomas characteristically show a well-circumscribed, mixed-density mass primarily occupying the anterior mediastinum (80%). Isolated posterior or middle mediastinal location is very rare and reported in only 2%-8% of cases [4]. CT findings of an intrapulmonary teratoma reveal a heterogeneous, lobulated intraparenchymal lung mass that contains mixed tissue types (soft tissue, fluid, or fat), with or without calcification, predominantly involving the left upper lobe, followed by the right upper and middle lobes. It is extremely rare in the lower lobes [6]. Calcification may be present in 26% of cases [7]. The heterogeneous internal composition may also produce a fat-fluid level or occasionally an air-fluid level mimicking cavitation when a bronchial communication has formed [4].

Despite these suggestive imaging features, the differential diagnosis of an intrapulmonary mass with similar characteristics and symptoms remains broad. Infectious causes must be carefully excluded, including fungal masses, complicated lung abscesses, and hydatid cysts. Primary lung cancer or an atypical malignant tumor should also be considered. In one reported case, demonstration of an aberrant systemic vascular supply raised the possibility of pulmonary sequestration, which was likewise managed with preoperative embolization [3].

Prognosis depends chiefly on the presence or absence of immature elements and on patient age. Approximately 80% of teratomas are mature; after complete resection, their prognosis is excellent, and no recurrences have been reported in the literature. In contrast, immature forms occurring in adults tend to follow a more aggressive course with a poorer prognosis. Less commonly, a mature teratoma may give rise to a carcinomatous, sarcomatous, or malignant germ-cell component, a condition then termed “malignant teratoma” or “teratocarcinoma” [7], which can lead to death within a few months through local extension or distant metastasis. This potentially unfavorable evolution justifies systematic excision of any mass suspected to be a teratoma, and chemotherapy may improve survival in certain metastatic forms [4].

## Conclusions

The rare primary intrapulmonary mature teratoma involving the left lower lobe described in this instance appeared as a calcified lung mass that resembled a pulmonary hamartoma. With histology demonstrating fully differentiated tissues and no immature or malignant elements, complete surgical excision proved to be both diagnostic and curative. Although long-term clinical and tumor-marker surveillance is still necessary, the prognosis following full excision is favorable. This case report emphasizes the importance of considering intrapulmonary teratoma in the differential diagnosis of an isolated calcified lung lesion.
